# Clinical Impacts of Allograft Biopsy in Renal Transplant Recipients 10 Years or Longer After Transplantation

**DOI:** 10.3389/ti.2024.13022

**Published:** 2024-07-18

**Authors:** Tomoko Namba-Hamano, Takayuki Hamano, Yohei Doi, Atsuko Hiraoka, Hiroaki Yonishi, Shinsuke Sakai, Atsushi Takahashi, Masayuki Mizui, Shigeaki Nakazawa, Kazuaki Yamanaka, Yoichi Kakuta, Ryoichi Imamura, Norio Nonomura, Yoshitaka Isaka

**Affiliations:** ^1^ Department of Nephrology, Osaka University Graduate School of Medicine, Suita, Japan; ^2^ Department of Nephrology, Nagoya City University Graduate School of Medical Sciences, Nagoya, Japan; ^3^ Department of Urology, Osaka University Graduate School of Medicine, Suita, Japan; ^4^ Department of Urology, Nagasaki University Graduate School of Biomedical Sciences, Nagasaki, Japan

**Keywords:** allograft biopsy, Banff score, eGFR slope, graft function, pathology, kidney transplantation

## Abstract

We aimed to investigate the clinical value of allograft biopsy performed long after renal transplantation. We retrospectively evaluated 99 allograft biopsies in recipients with transplantation vintages of 10 years or longer. Mixed-effects model showed that 1-year estimated glomerular filtration rate (eGFR) slopes after biopsy were significantly greater than those before biopsy [−3.13, −4.42 mL/min/1.73 m^2^/year, *p* = 0.01]. Renal biopsy changed the treatment strategies in more than half of the patients. Improvement in eGFR slopes was pronounced in 51 patients with treatment modification based on the biopsy results [2.27 (95% confidence interval (CI): 0.66, 3.89) mL/min/1.73 m^2^/year], whereas no improvement was observed in those without [0.33 (95% CI: −1.05, 1.71) mL/min/1.73 m^2^/year, P_interaction_ = 0.001]. Among the treatment modifications, enhancement of immunosuppression (IS) led to the most remarkable improvement in eGFR slope. Patients with g scores ≥2 were more likely to receive IS enhancement than those with g scores = 0 [odds ratio; 15.0 (95% CI: 1.65, 136)]. Patients with active glomerulitis (g ≥ 1) without chronicity (cg ≤ 1) showed the most significant improvement in eGFR slope. Given the prevalence of active glomerulitis (g ≥ 1, 21%), which is responsive to treatment even long after transplantation, and the observed magnitude of eGFR slope improvement, renal biopsy can indeed improve allograft prognosis.

## Graphical Abstract

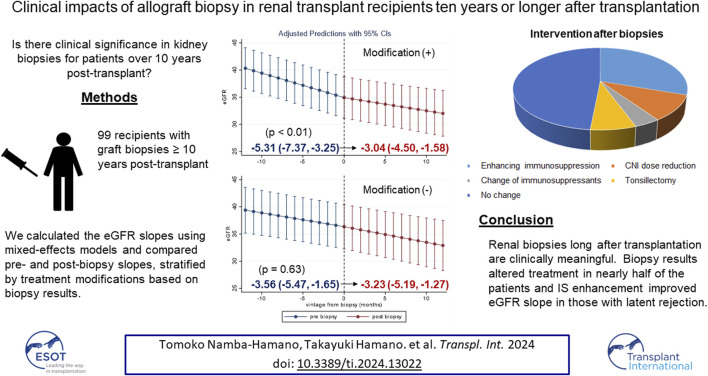

## Introduction

Introduction of novel immunosuppressive agents has significantly improved graft outcomes in kidney transplant recipients (KTRs) since the 1980s. While the 1-year graft survival rate is over 90%, more than 10% of KTRs in the US and European countries lose their graft function by 5 years post-transplantation. Therefore, improving the long-term graft survival rate remains challenging [1, 2]. Furthermore, allograft kidney dysfunction is caused by immunological factors, such as rejection, and non-immunological factors, such as hypertension, glucose intolerance, and donor age [3]. In addition, allograft kidney dysfunction can be driven by conditions unique to transplantation, such as drug toxicity, infection, and recurrent nephritis [4].

Allograft renal biopsy plays an important role in differentiating between these conditions. In particular, indication biopsy plays an essential role in diagnosing rejection in an early phase. Immunosuppressive therapy for early rejection diagnosed by biopsy led to better graft outcomes subsequently, especially in patients in whom early intervention recovered the renal function to near baseline [5]. However, it remains unknown whether allograft biopsy long after transplantation can positively affect renal allografts through treatment modification. Although the risk of T cell-mediated rejection was reported to be relatively low in long-term transplanted grafts, the causes of graft loss were revealed to be multifactorial and more complex than expected previously [6]. Allograft biopsy performed long after transplantation might contribute to diagnosing its etiology, thus improving graft outcomes. However, there have been few reports focusing on renal biopsies performed at 10 years or longer after transplantation. Therefore, we aimed to examine the clinical impacts of renal biopsies in those transplant recipients. Specifically, we addressed the following three issues: 1) whether indication biopsy, performed more than 10 years after transplantation, improves graft prognosis, 2) whether the treatment changes based on renal biopsy results could lead to improvement in renal function after renal biopsy, and 3) the pathological findings associated with an eGFR improvement. In this study, since most patients were proteinuria-negative, we used eGFR slope [7] instead of urine albumin-to-creatinine ratio (UACR) reduction as a surrogate outcome.

## Patients and Methods

### Study Design and Population

We conducted a multicenter retrospective observational study at Osaka University Hospital, Inoue Hospital Attached Clinic, and Takatsuki General Hospital in Osaka, Japan. We enrolled KTRs, who had undergone indication biopsies of allografts between March 2002 and January 2019 and had transplantation vintages of 10 years or longer at the biopsy.

In patients who underwent multiple biopsies more than 10 years post-transplant, the first biopsy conducted after 10 years was used for analysis. We excluded the patients who received additional immunosuppressive therapy immediately before renal biopsy. The study was conducted in accordance with the principles of the Declaration of Helsinki. The Ethics Committee of Osaka University Hospital approved the study and waived the need for informed consent because of the retrospective study design (approval number: 17334-3). In addition, we provided the patients with the option to opt out of participation whenever during the study.

### Data Collection

Patient characteristics at the time of graft biopsy were collected as baseline data. In addition, information regarding transplantation, including transplantation vintage, donor characteristics (age at transplantation, sex, and living or cadaveric donor), simultaneous pancreas-kidney transplantation, immunological factors (ABO compatibility and human leukocyte antigen (HLA)-matching status), and dialysis vintage before transplantation, was collected. According to the Banff 2017 classification [8], except for i-IFTA, biopsy samples were re-scored by a renal pathologist (T.N.). The i-IFTA score, a score of the chronic active T-cell-mediated rejection component, was evaluated retrospectively according to Banff 2019 [9]. A microvascular inflammation (mvi) score was defined as a combination of the glomerulitis score (g) and peritubular capillaritis score (ptc) according to the Banff 2013 classification [10]. The eGFRs were calculated using the following Japanese standard formula: 194 × creatinine^−1.094^ × age^−0.287^ (if female, ×0.739) [11].

### Statistical Analyses

Data are presented as numbers (percentages) for categorical variables, means (standard deviations) for normally distributed variables, or medians (interquartile ranges [IQR]) for skewed variables. We compared the baseline characteristics between KTRs with and without treatment modification based on the biopsy results. The means of normally distributed variables and the proportion of each category were compared using the Student’s t-test and Fisher’s exact test, respectively. The Wilcoxon rank-sum test was used to compare the distributions of categorical variables. Differences in continuous variables across groups were tested using a one-way analysis of variance (ANOVA).

The study outcome was the improvement in 1-year eGFR slope after allograft biopsy, defined as (post-biopsy eGFR slope–pre-biopsy eGFR slope). For pre-biopsy and post-biopsy eGFR slope calculations, we used the eGFR data of the preceding 1 year before the index biopsy and the 1-year follow-up data after biopsy. We separately estimated the eGFR slopes for each individual in the pre- and post-biopsy periods using linear mixed-effects models. Random intercepts and time slopes were included to determine the eGFR trajectory in these models. As a primary analysis, we compared the eGFR slopes for the two periods using paired t-tests in all patients and in those stratified by treatment modification after biopsy. For sensitivity analysis, we created a linear mixed-effects model with time-dependent eGFR as the dependent variable to investigate whether eGFR improvement after biopsy differed between patients with and without treatment modifications. In this model, we included a 3-way interaction term among continuous-time, categories (pre-and post-biopsy), and treatment modification in addition to continuous-time, categories, and treatment modifications as fixed effects. The two-way interaction between time and the pre- or post-biopsy period would explain whether the effect of time on eGFR (eGFR slope) was different across biopsy periods. The addition of the 3-way interaction term to the model allowed us to explore whether treatment modification modifies eGFR slope difference across the biopsy period.

Logistic regression models were employed to analyze the Banff scores associated with IS enhancement. Covariates in the multivariate analysis included sex, recipient age, and eGFR at the time of biopsy.

Statistical significance was set at *p* < 0.05. All the statistical analyses were performed using Stata version 16 (StataCorp, College Station, TX, United States).

## Results

### Clinical Characteristics of Participants

**FIGURE 1:**
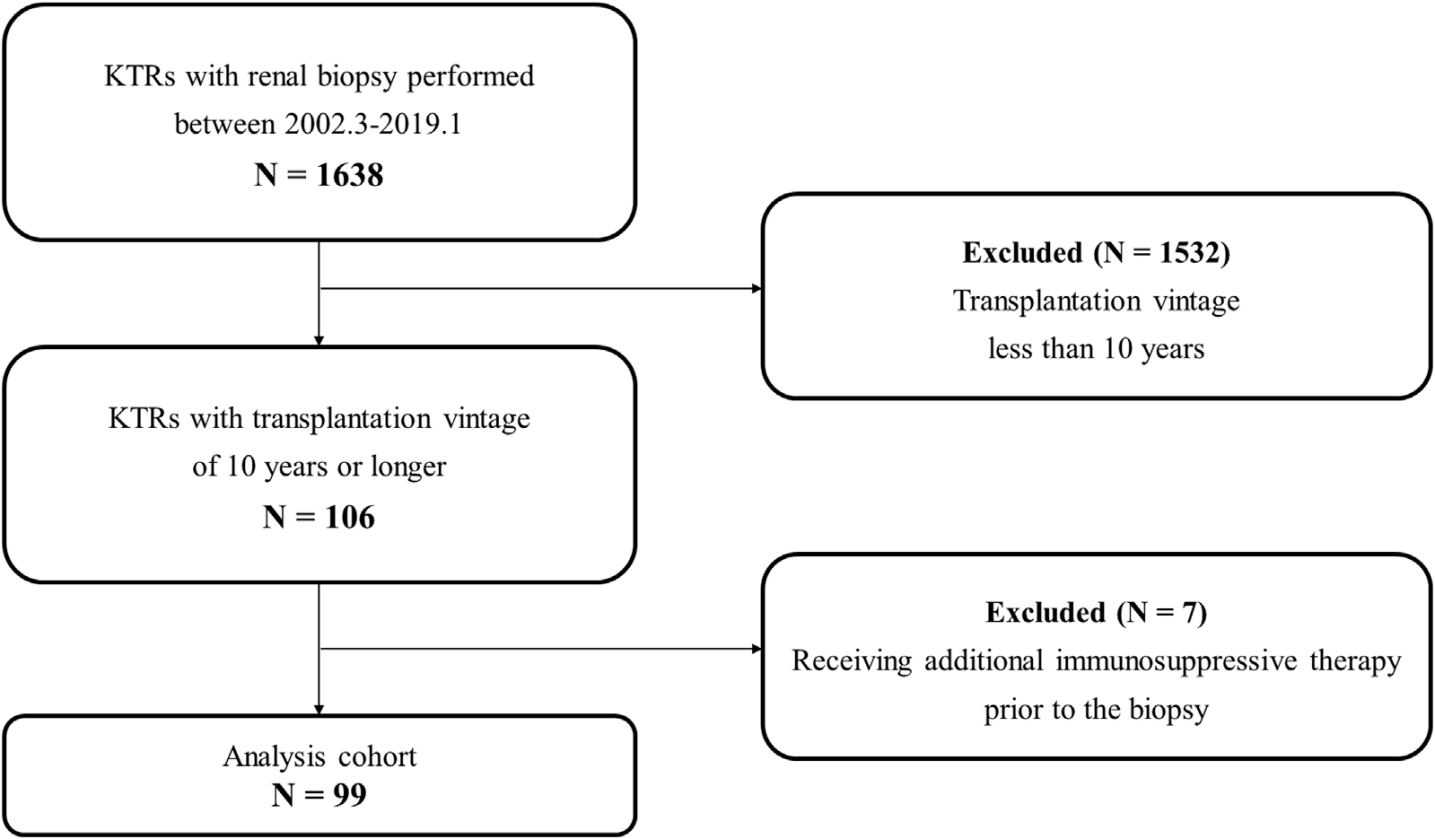
Flow diagram of the study population. Abbreviations: KTR, kidney transplant recipient.

Figure 1 shows a flowchart of the participant selection process. Between March 2002 and January 2019, 1,638 patients underwent allograft biopsy. Among them, 106 had a transplantation vintage of 10 years or longer. In addition, seven patients received additional IS for probable rejection just before the biopsy. Therefore, 99 patients were eligible for this study based on all the exclusion criteria.

Table 1 shows the baseline characteristics of all the 99 patients. The median age, eGFR, and proteinuria at biopsy were 50 years, 34.8 mL/min/1.73 m^2^, and 0.49 g/day, respectively. The median transplantation vintage at biopsy was 13.7 years. Of the 99 patients, 78 (79%) underwent living-donor kidney transplantation. More than half of the living donors were recipients’ mothers (52.6%), followed by their fathers (14.1%). The remainder, except for one case, were kidney transplants from deceased donors, five of which were simultaneous pancreas-kidney transplants. There were five (5.1%) ABO-incompatible transplants. Ninety percent of the patients received steroid-based IS supplemented with calcineurin inhibitors (CNI), mostly cyclosporine A (CsA). The mean trough levels of cyclosporin users were 62.3 ± 17.8 ng/mL, and that of tacrolimus users were 3.04 ± 1.20 ng/mL. Mycophenolate mofetil (MMF) was used most commonly as an antimetabolic agent. Sixteen patients had been diagnosed with acute rejection prior to this study. All cases were of acute cellular rejection, and no patients had acute humoral rejection. No cases had been diagnosed with BK virus-associated nephropathy among the subjects included in the analysis.

**TABLE 1:** Clinical characteristics of patients.

	Total	Without treatment modification*	With treatment modification**	*p*-value
Recipient information				
Number of patients	99	48	51	
Biopsy time from TPL (year)	13.7 (11.4, 19.3)	14.8 (11.8, 19.3)	12.9 (10.7, 19.3)	0.16
Dialysis vintage (year)	1.7 (0.8, 5.6)	2.3 (0.7, 6.4)	1.3 (0.9, 3.9)	0.33
Male gender (%) (n)	64 (63)	67 (32)	61 (31)	0.54
Age at biopsy (year)	50 ± 11	53 ± 12	47 ± 11	<0.01
Prior biopsy-proven acute rejection (%) (n)	6.2 (16)	12.5 (6)	19.6 (10)	0.42
Indication for biopsy (%) (n)				
Decline of eGFR	47 (48)	21 (44)	26 (51)	0.60
Increase in urinary protein	33 (33)	15 (31)	18 (35)	
Both	8 (8)	5 (10)	3 (6)	
Others	11 (11)	7 (15)	4 (8)	
eGFR at biopsy (mL/min/1.73 m^2^)	34.8 ± 15.0	35.2 ± 14.5	34.4 ± 15.5	0.79
Urinary protein excretion at biopsy (g/day)	0.49 (0.18, 0.89)	0.45 (0.16, 0.8)	0.49 (0.20, 1.02)	0.38
Systolic blood pressure at biopsy (mmHg)	125 ± 16	126 ± 17	125 ± 16	0.8
Diastolic blood pressure at biopsy (mmHg)	77 ± 10	77 ± 10	78 ± 11	0.54
HbA1c at biopsy (%)	5.47 ± 0.78	5.48 ± 0.75	5.47 ± 0.80	0.95
Donor information				
Donor age at TPL (year)	50 ± 13	51 ± 15	49 ± 11	0.45
Gender of donors (male), n (%)	23 (28)	13 (33)	10 (23)	0.31
Deceased donation, n (%)	20 (20)	11 (24)	9 (18)	0.46
Relationship of living donors, n (%)				
Mother	41 (53)	16 (44)	25 (60)	0.60
Father	11 (14)	5 (14)	6 (14)	
Sister	8 (10)	6 (17)	2 (5)	
Spouse	8 (10)	4 (11)	4 (10)	
Brother	6 (8)	3 (8)	3 (7)	
Others	4 (5)	2 (6)	2 (5)	
Simultaneous pancreas transplantation, n (%)	5 (5.1)	4 (8.3)	1 (2.0)	0.20
ABO incompatibility, n (%)	5 (5.1)	2 (5.9)	3 (7.1)	1.00
HLA mismatch				
A (0, 1, 2)	27, 33, 2	9, 20, 0	18, 13, 2	0.04
B (0, 1, 2)	15, 44, 3	8, 20, 1	7, 24, 2	0.91
DR (0, 1, 2)	13, 46, 1	7, 21, 0	6, 25, 1	0.87
Average HLA mismatch	2.1 ± 1.1	2.2 ± 1.0	2.1 ± 1.1	0.59
Immunosuppressants, n (%)				
Corticosteroid	90 (91)	45 (94)	45 (88)	0.49
Cyclosporine A	55 (56)	25 (52)	30 (59)	0.55
Tacrolimus	40 (40)	20 (42)	20 (39)	0.84
Mycophenolate mofetil	53 (54)	24 (50)	29 (57)	0.55
Azathioprine	21 (21)	9 (19)	12 (24)	0.63
Mizoribine	15 (12)	10 (21)	5 (10)	0.16
Antihypertensives, n (%)				
Angiotensin-converting-enzyme inhibitor	28 (28)	11 (23)	17 (33)	0.27
Angiotensin II receptor blocker	62 (63)	26 (54)	36 (71)	0.10
Calcium channel blocker	47 (47)	25 (52)	22 (43)	0.42
Diuretics	20 (20)	9 (19)	11 (22)	0.81
β blocker	21 (21)	9 (19)	12 (24)	0.43
Mineral corticoid receptor antagonist	7 (7)	3 (6)	4 (8)	1.00

Abbreviations: TPL, transplantation; eGFR, estimated glomerular filtration ratio; HLA, human leukocyte antigen. * denotes patients without treatment modification, while ** denotes patients with treatment modification. *p*-value for the difference between patients with treatment modification and those without.

In nearly half of the patients (51%), the doctors in charge changed their treatment strategies based on the biopsy diagnosis. Patients were divided into two groups according to treatment modification. Baseline characteristics of both groups were similar, except for age (Table 1). Details of the treatment modifications are described as follows. Enhancing immunosuppressive therapy was the most common treatment modification (30 patients; 58.8%), followed by a reduction in CNI doses in 9 patients (19.6%) and a change in immunosuppressive agents in 4 patients (7.8%). Enhancing immunosuppressive therapy included methylprednisolone pulse therapy, 15-deoxyspergualin, and increased doses of immunosuppressive agents. As a methylprednisolone pulse therapy, we administered methylprednisolone sodium succinate for 3 consecutive days in 12 patients. 15-deoxyspergualin was administered for 7 successive days per course. The usual number of treatment cycles was 5 or 6 courses, with an average dosage of 4.9 mg/kg per administration in 9 patients. As an immunosuppression enhancement, increased doses of tacrolimus (2 patients), prednisone (2 patients), cyclosporine (1 patient), azathioprine (1 patient), and mycophenolate mofetil (1 patient) were administered (Supplementary Table S1). These patients had their immunosuppressive medication increased by 2.25 times compared to before the biopsy. Everolimus (1.5 mg/day) was administered to two patients. Seven (13.8%) patients were diagnosed with active IgA nephropathy, and subsequently underwent tonsillectomy.

### Histological Findings of Biopsy Samples

Among the acute Banff scores, few patients had positive i- and t-scores. All the patients had v scores of zero (Table 2). Regarding chronic Banff scores, the ci, ct, and cg scores were positive in 55%, 65%, and 39% of patients, respectively. Only few patients had positive cv scores. Notably, the ah scores, indicating hyalinosis of the small arteries, and the aah scores, implying CNI toxicity, were positive in most cases (92% and 80%, respectively). In univariate analysis, the treatment modification group had significantly higher ti scores than the no-modification group (Table 2). We found no difference in other scores, including the i-IFTA score, between the two groups. The breakdown of glomerular lesions (IgA nephropathy, diabetic nephropathy, and membranous glomerulopathy) is shown in Supplementary Figure S1; there was no significant difference between the two groups (*p* = 0.64). A substantial percentage of enrolled patients did not undergo C4d staining.

**TABLE 2:** Distribution of each Banff score stratified by treatment modification based on biopsy results.

A. Active lesions				B. Chronic lesions				C. Acute and chronic Banff scores and arterial scores			
	Without treatment modification*	With treatment modification**	*p*-value		Without treatment modification*	With treatment modification**	*p*-value		Without treatment modification*	With treatment modification**	*p*-value
*i* score	n (%)	n (%)		*ci* score	n (%)	n (%)		*ti* score	n (%)	n (%)	
0	43 (90)	46 (90)	1.00	0	23 (48)	22 (43)	0.85	0	39 (81)	32 (63)	0.04
1	5 (10)	5 (10)		1	18 (38)	22 (43)		1	7 (15)	18 (35)	
2	0 (0)	0 (0)		2	7 (14)	6 (11)		2	2 (4)	1 (2)	
3	0 (0)	0 (0)		3	0 (0)	1 (2)		3	0 (0)	0 (0)	

*t* score				*ct* score				*i-IFTA* score			
0	46 (96)	49 (96)	1.00	0	19 (40)	16 (31)	0.57	0	24 (50)	23 (45)	0.36
1	2 (4)	2 (4)		1	25 (52)	32 (63)		1	14 (29)	14 (27)	
2	0 (0)	0 (0)		2	4 (8)	3 (6)		2	7 (15)	5 (10)	
3	0 (0)	0 (0)		3	0 (0)	0 (0)		3	3 (6)	9 (18)	

*g* score				*cg* score				*ah* score			
0	42 (89)	36 (72)	0.15	0	32 (70)	28 (56)	0.11	0	3 (6)	5 (10)	0.80
1	4 (9)	9 (18)		1	8 (17)	5 (10)		1	12 (26)	11 (22)	
2	1 (2)	4 (8)		2	1 (2)	3 (6)		2	21 (45)	25 (50)	
3	0 (0)	1 (2)		3	5 (11)	14 (28)		3	11 (23)	9 (18)	

*v* score				*cv* score				*aah* score			
0	47 (100)	49 (100)		0	46 (98)	47 (96)	1.00	0	12 (26)	9 (18)	0.77
1	0 (0)	0 (0)		1	1 (2)	2 (4)		1	7 (14)	6 (12)	
2	0 (0)	0 (0)		2	0 (0)	0 (0)		2	16 (34)	20 (40)	
3	0 (0)	0 (0)		3	0 (0)	0 (0)		3	12 (26)	15 (30)	

*ptc* score				*mm* score							
0	37 (77)	33 (65)	0.19	0	39 (85)	36 (72)	0.29				
1	8 (17)	7 (14)		1	6 (13)	10 (20)					
2	1 (2)	5 (10)		2	1 (2)	1 (2)					
3	2 (4)	6 (11)		3	0 (0)	3 (6)					

* denotes patients without treatment modification, while ** denotes patients with treatment modification. *p*-value for the difference between patients with treatment modification and those without.

### One-Year eGFR Slopes Stratified by the Presence of Treatment Modification

**FIGURE 2:**
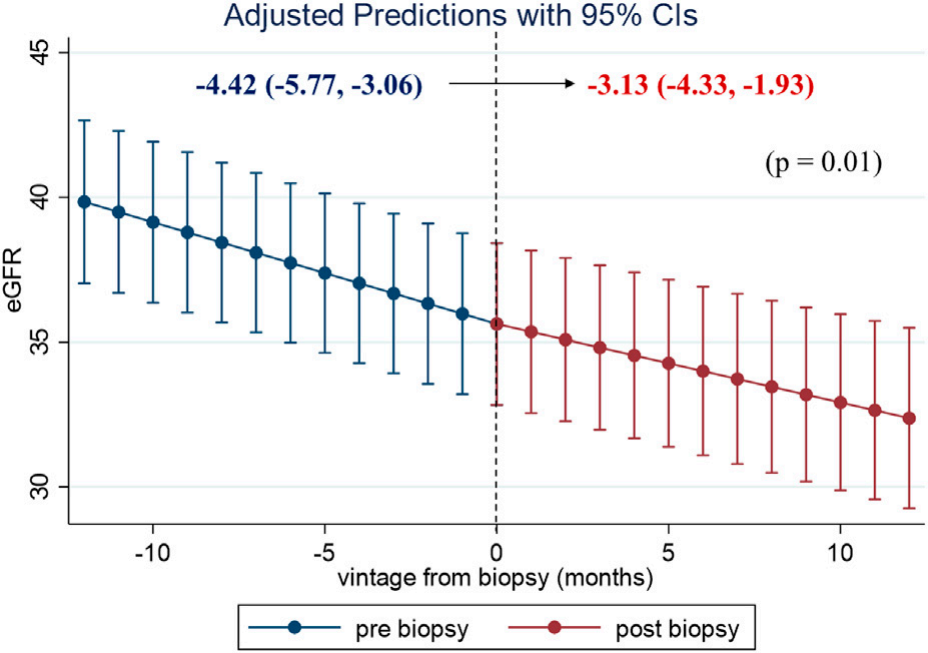
One-year eGFR trajectory, pre- and post-biopsy, with slopes for all the patients. The eGFR level with 95% confidence interval (CI) at each time point in the 99 patients was estimated using a mixed-effects model. A significant improvement in eGFR slope was observed after the index biopsy. Abbreviations: eGFR, estimated glomerular filtration rate; CI, confidence interval.

**FIGURE 3:**
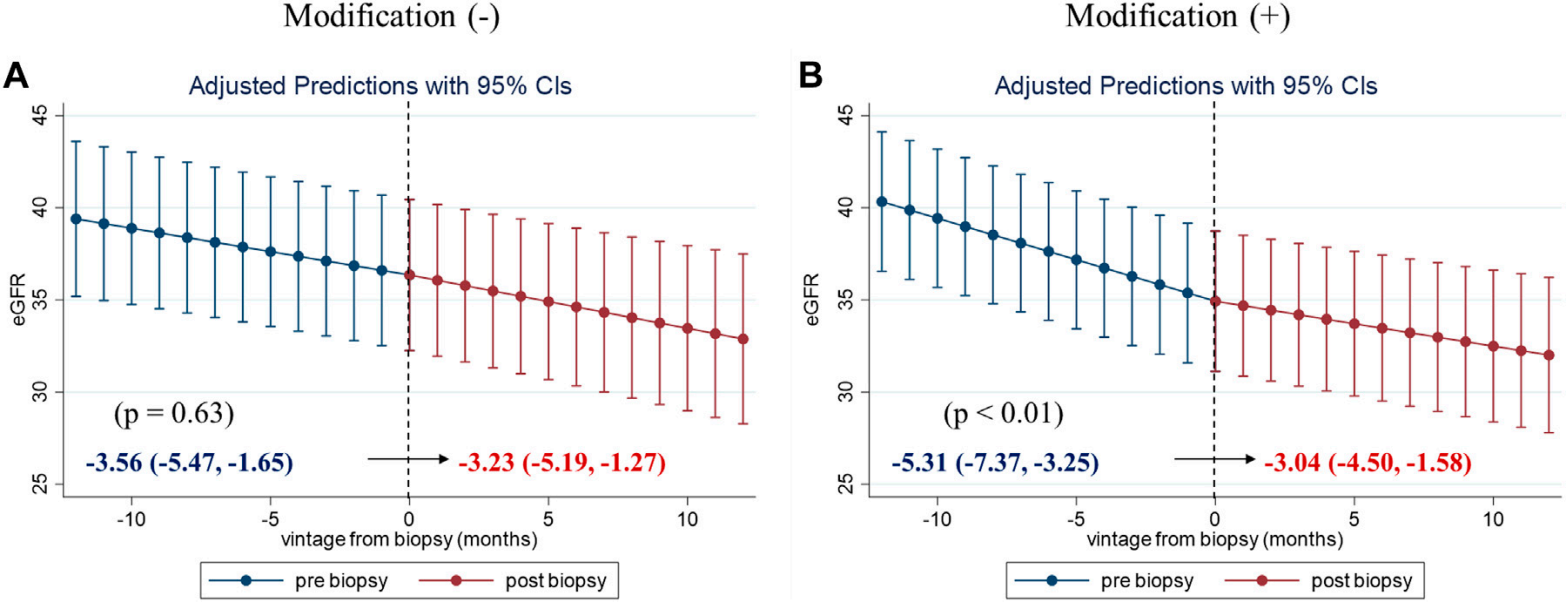
One-year eGFR trajectory, pre- and post-biopsy, with slopes stratified by the treatment modification. One-year eGFR slopes for patients without treatment modification **(A)** and for those with treatment modification **(B)**. The magnitude of eGFR slope improvement was significantly pronounced in patients with treatment modification [2.27 (95% CI: 0.66, 3.89)]. Abbreviations: eGFR, estimated glomerular filtration ratio; CI, confidence interval.

To evaluate the clinical impact of graft biopsy, we compared the eGFR slope 1 year before and after the biopsy (Figure 2). The median number of serum creatinine measurements recorded per patient was 31 during the 2 years [interquartile range (IQR): 26–35]. The 1-year eGFR slope before biopsy for all the 99 patients was −4.42 mL/min/1.73 m^2^/year [95% confidence interval (CI): −5.77, −3.06], and the eGFR slope after the biopsy was −3.13 mL/min/1.73 m^2^/year (95% CI: −4.33, −1.93). A significant improvement was observed in eGFR slope after the biopsy [1.28 mL/min/1.73 m^2^/year (95% CI: 0.29, 2.27), *p* = 0.01]. Stratified analyses were performed based on the treatment modifications. Among the patients with no treatment modification, we did not observe significant improvement in the 1-year eGFR slope [−3.56 mL/min/1.73 m^2^/year (95% CI: −5.47, −1.65) before biopsy, −3.23 mL/min/1.73 m^2^/year (95% CI: −5.19, −1.27) after biopsy]. On the other hand, eGFR slope significantly improved in patients with treatment modification [−5.31 mL/min/1.73 m^2^/year (95% CI: −7.37, −3.25) before biopsy, −3.04 mL/min/1.73 m^2^/year (95% CI: −4.50, −1.58) after biopsy, *p* < 0.01] (Figure 3). Even after censoring at eGFR less than 15, a significant improvement in the eGFR slope was observed among patients with treatment modification [−5.01 mL/min/1.73 m^2^/year (95% CI: −6.64, −3.38) before biopsy, −3.17 mL/min/1.73 m^2^/year (95% CI: −3.49, −0.20) after biopsy, *p* = 0.03]. In contrast, the patients without treatment modification showed no significant change in eGFR slope [−3.57 mL/min/1.73 m^2^/year (95% CI: −4.84, −2.32) before biopsy, −3.07 mL/min/1.73 m^2^/year (95% CI: −4.47, −1.67) after biopsy]. In other words, the magnitude of eGFR slope improvement was significantly pronounced in patients with treatment modification [2.27 mL/min/1.73 m^2^/year (95% CI: 0.66, 3.89)] than in patients without [0.33 mL/min/1.73 m^2^/year (95% CI: −1.05, 1.71)]. For sensitivity analysis, we employed a mixed-effects model using all data collected during the 2 years. A 3-way interaction term among the continuous-time, categories (pre-and post-biopsy), and treatment modification was significant in this model (*p* = 0.001). This indicated that eGFR slope changes were affected by treatment modification.

### Difference in eGFR Slope Improvement Based on the Types of Treatment Modifications and Indications of Biopsy

**FIGURE 4:**
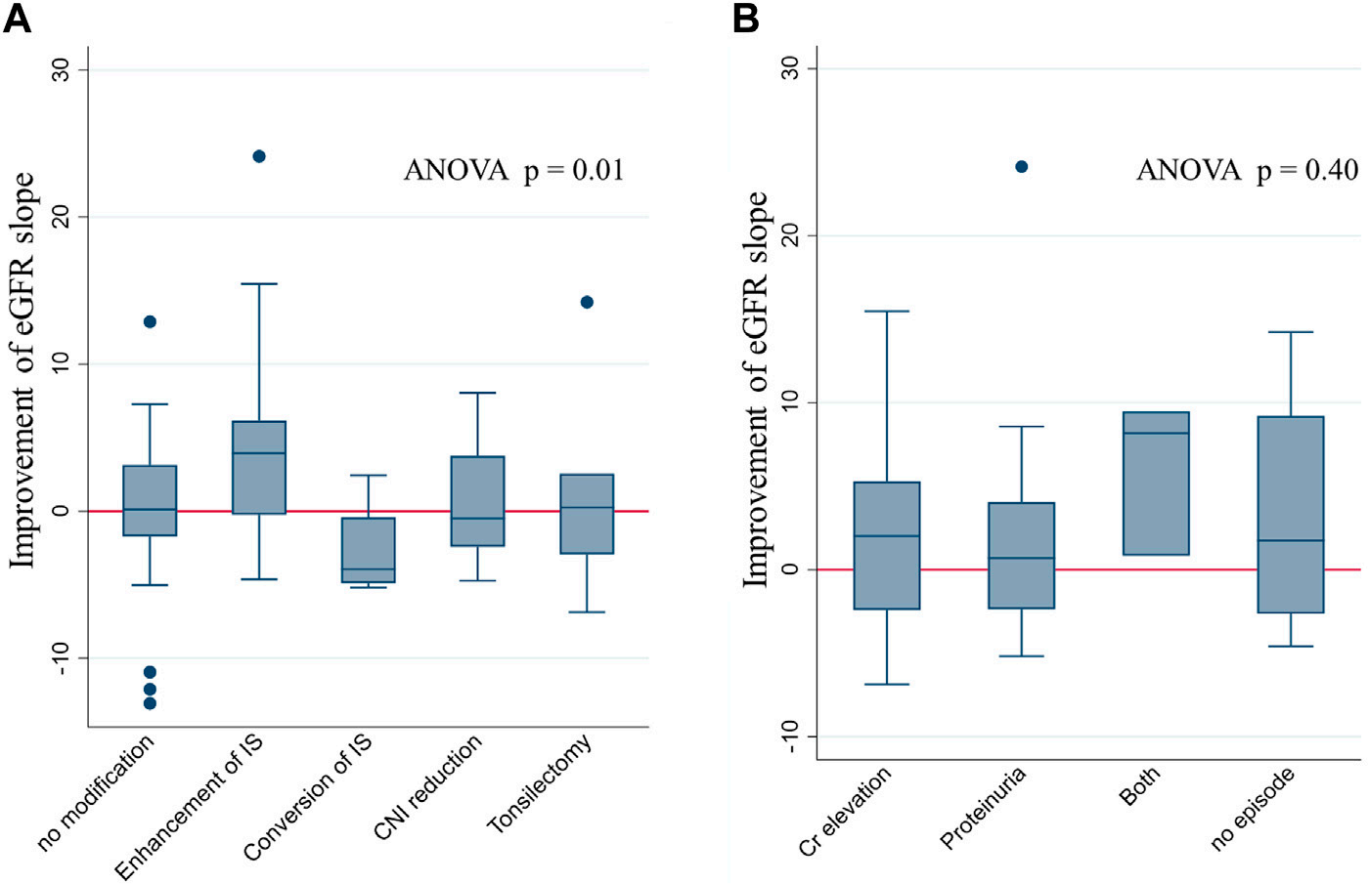
eGFR slope improvement after the index biopsy stratified by types of modification and indications of biopsy. eGFR slope improvement after biopsy, stratified by the type of modification **(A)** and the indications for biopsy **(B)**. Improvement in eGFR was most prominent in patients with IS enhancement. No significant difference in the eGFR slope change was found among the groups stratified according to biopsy indications. Abbreviations: eGFR, estimated glomerular filtration rate; ANOVA, analysis of variance; IS, immunosuppression; CNI, calcineurin inhibitor.

The differences in eGFR slope change, before and after biopsy, were compared across the types of treatment modifications. The most remarkable improvement in eGFR slope after the biopsy was observed in patients whose IS was enhanced based on the biopsy results (including methylprednisolone pulse therapy, 15-deoxyspergualin administration, and an increase in IS dosage). An improvement in eGFR slope was observed in some, but not all, patients who underwent tonsillectomy or had their CNI doses reduced (Figure 4A). Supplementary Figure S2 shows the eGFR trajectories in representative cases of eGFR improvement after IS enhancement (nine cases). Among them, eight patients underwent methylprednisolone pulse therapy. In three patients with increased CNI doses after biopsy, post-biopsy trough levels of CNI were 1.6 times higher than their pre-biopsy levels. Their eGFR slope ameliorated from −3.66 mL/min/1.73 m^2^/year (95% CI: −13.6, 6.31) before biopsy to −0.24 mL/min/1.73 m^2^/year (95% CI: −9.99, 9.52) after biopsy. Unfortunately, the sample size was too small to detect a significant difference.

In contrast, there was no significant difference in eGFR slope changes among the various indications for biopsy (Figure 4B).

### Pathological Findings Related to Antibody-Mediated Rejection Prompted IS Enhancement

**FIGURE 5:**
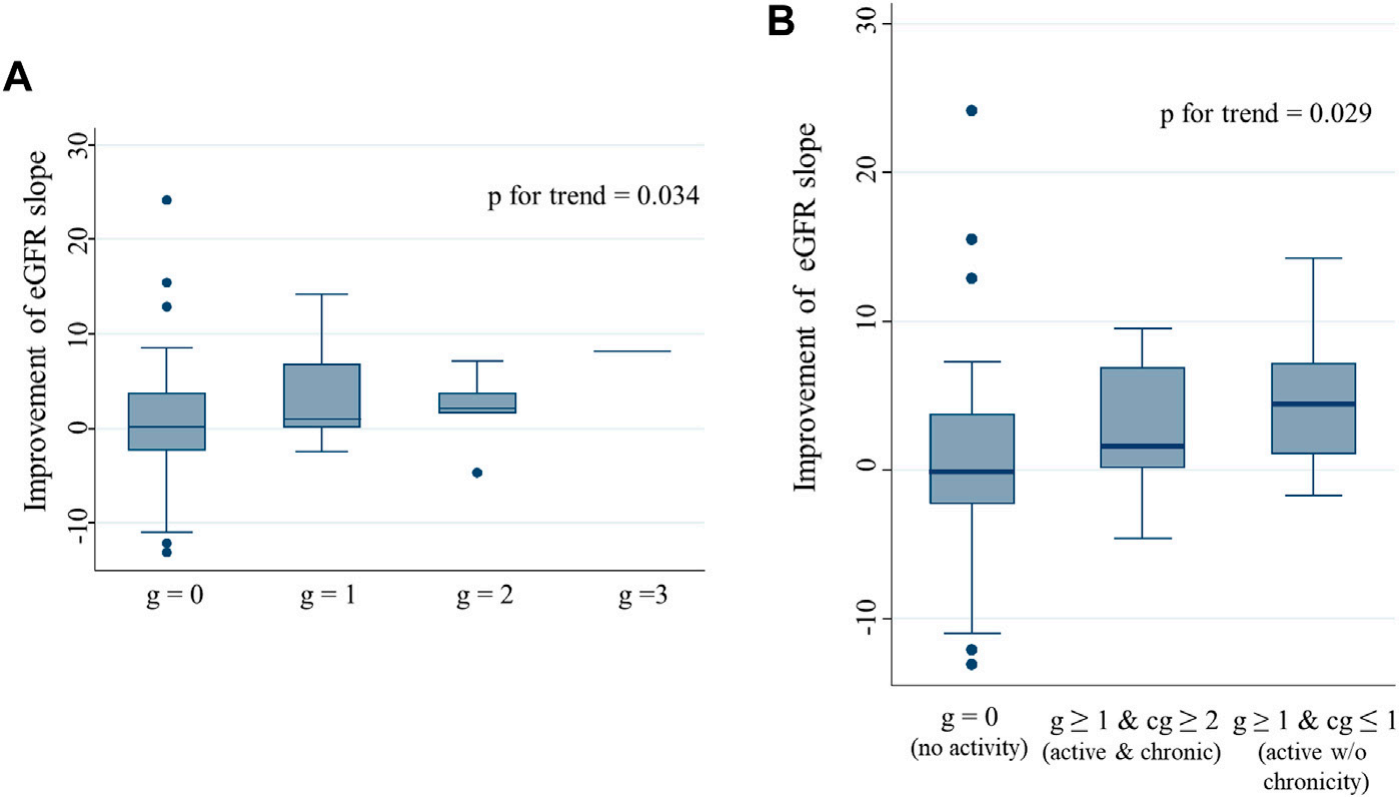
eGFR slope improvement after biopsy stratified by g and cg scores Improvement in eGFR slope after biopsy in patients, stratified by g score **(A)**, and by combination of g and cg scores **(B)**. A significant positive trend was observed between the g scores and eGFR slope improvement after biopsy. Patients with active glomerulitis showed greater improvement in the eGFR slope after biopsy than those without. Among patients with active glomerulitis, a more prominent improvement in the eGFR slope was found in patients without chronicity than in those with. Abbreviations: eGFR, estimated glomerular filtration rate.

**FIGURE 6:**
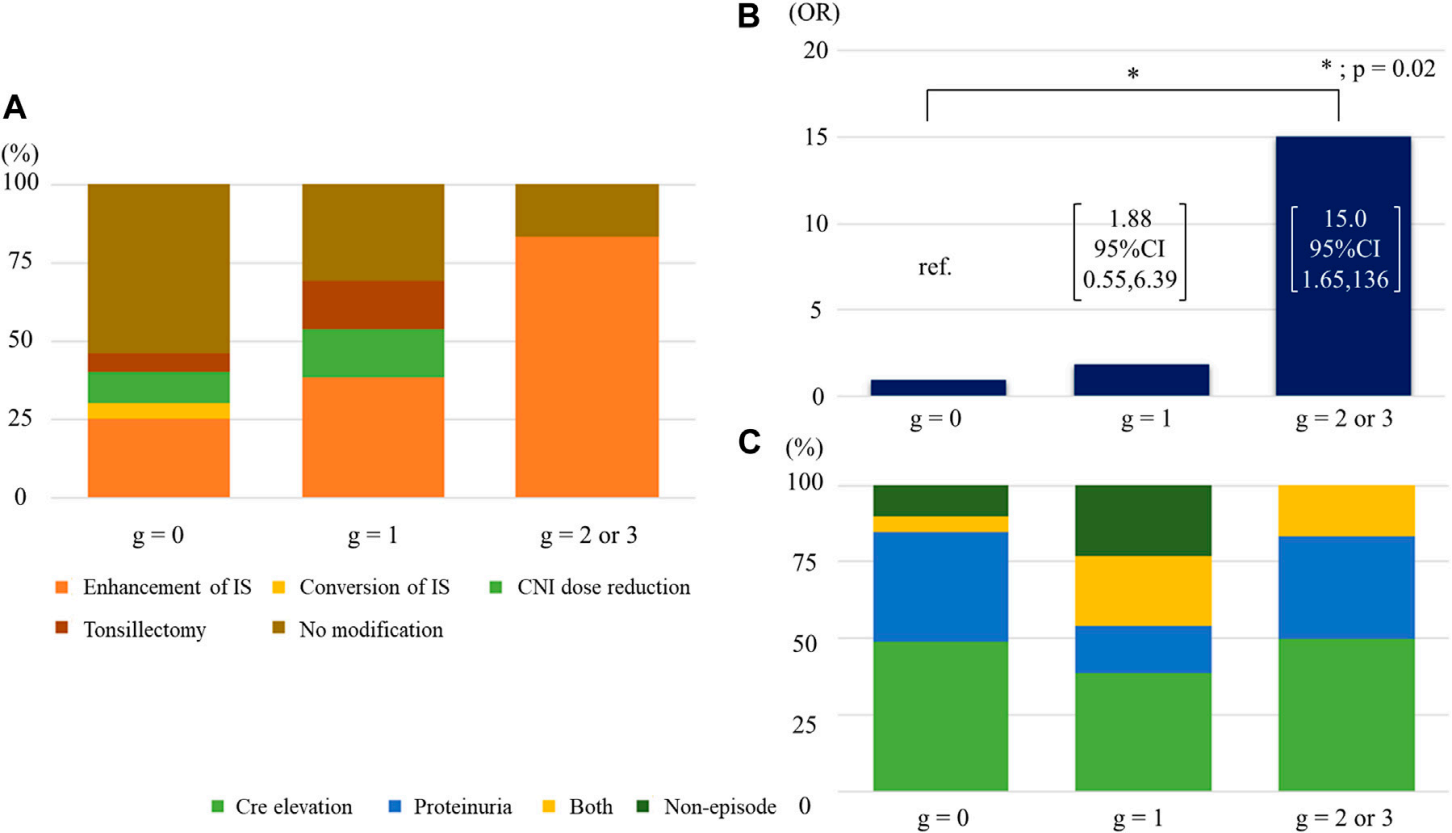
Association of g score with IS enhancement **(A)** Breakdown of treatment modifications by g scores. **(B)** The odds ratios of receiving increased immunosuppressants across g scores. **(C)** Breakdown of indications of biopsy by g scores. Abbreviations: CNI, calcineurin inhibitor; IS, immunosuppression; OR, odds ratio; Cre, creatinine.

IS enhancement was found to be more effective for improving eGFR slope than the other interventions. We compared the Banff scores between patients with IS enhancement and those without. In univariate analysis, patients with IS enhancement had significantly higher g, ptc, and cg scores than those without (Supplementary Figure S3). The three scores were correlated; higher g-scores were associated with a higher percentage of patients with positive ptc and cg scores (Supplementary Figures 4A, B). Patients with higher cg scores were more likely to have positive ptc and mvi (g + ptc) scores ≥ 2 (Supplementary Figures S4C, D). Half of the patients (48.3%) with the enhancement of immunosuppressants had mvi score of 2 or higher. Among the four parameters, only the g-score showed a significant positive trend, with eGFR slope improvement after biopsy (Figure 5A). With an increase in the g-score, the proportion of patients with IS enhancement increased, whereas the proportion of patients without treatment modifications decreased (Figure 6A). The group with g scores ≥2 showed an odds ratio as high as 15.0 (95% CI: 1.65, 136), compared to the group with a g score of zero (Figure 6B). Regarding biopsy indications, the proportion of creatinine elevation did not show any difference (approximately 50%) among the three groups stratified by g-scores (Figure 6C).

To explore the impact of activity and chronicity of glomerular changes on eGFR slope improvement, we divided the patients into three groups according to the combination of g and cg scores; 1) g = 0 and any cg score (no activity), 2) g ≥ 1 and cg ≥ 2 (active and chronic), and 3) g ≥ 1 and cg ≤ 1 (active without chronicity). Patients with active glomerulitis showed better improvement in eGFR slope than those without (g score = 0). In particular, patients with active glomerular lesions, but without chronicity, showed the greatest improvement in eGFR slope; a significant trend was observed (*p* = 0.029) (Figure 5B).

## Discussion

The following novel findings were obtained in this study: First, the eGFR slope showed significant improvement after renal biopsy in patients with a transplantation vintage of 10 years or longer. This indicated the clinical importance of indication biopsy even during the late phase post renal transplantation. Second, treatment modifications based on the biopsy results, especially IS enhancement, had the greatest impact on improving the eGFR slope. This implied that patients exhibit a state of alloimmunity, for which immunosuppressive agents are effective, even long after transplantation. Third, physicians are likely to increase IS in cases with allograft glomerulopathy or with active inflammation in the glomerulus and peritubular capillaries. Among the findings, only glomerulitis was associated with improved eGFR slope after biopsy. However, no association was observed between the indications for biopsy and the severity of glomerulitis. This indicated that the diagnosis of transplant glomerulitis could not be inferred from the clinical information before biopsy. We observed little differences in baseline characteristics between patients with and without treatment modifications. Moreover, no significant difference was found in eGFR slope changes among the various indications for biopsy. These results underscore the importance of renal biopsy in clinical decision-making, even in long-term transplant recipients.

Overall, this study showed that allograft biopsy plays an important role in patients with a transplantation vintage of 10 years or longer.

This is the first study demonstrating clinical consequences after treatment modifications based on long-term pathological findings after transplantation. The rate of eGFR decline, along with the change in albuminuria [12, 13], have been reported to be an excellent surrogate marker for assessing renal outcomes [14, 15]. To evaluate the effect of treatment modification on hard outcomes, such as kidney failure with replacement therapy, we selected the eGFR slope among various surrogate endpoints, instead of albuminuria change, since urinary protein was negative in most KTRs, except for those with recurrent nephritis. Moreover, the association between graft outcome and eGFR slope for at least 12 months after biopsy has been confirmed recently in KTRs [16]. This result supports the validity of eGFR slope as a surrogate endpoint for graft survival. In our study, the improvement in eGFR slope after biopsy was as noticeable as 2.27 mL/min/1.73 m^2^/year in the group with treatment modification. The value was clinically substantial, since a meta-analysis of clinical trials reported that a change in eGFR slope by 0.75 mL/min/1.73 m^2^/year corresponds to an average 27% lower hazard of kidney failure with replacement therapy [7, 15]. In Japan, the mean eGFR at re-initiation of dialysis after transplantation was reported to be 5.45 mL/min/1.73 m^2^ [17]. In this study, the mean eGFR level at biopsy, and the 1-year eGFR slope decline after biopsy, in patients with treatment modification, was 34.4 mL/min/1.73 m^2^ and 3.04 mL/min/1.73 m^2^/year, respectively; therefore, it was 9.5 years from the time of biopsy to the initiation of renal replacement therapy. On the other hand, if the patients had not received treatment modification based on biopsy results, it would have been 5.5 years after biopsy that they reached kidney failure with replacement therapy, based on the pre-biopsy eGFR slope (−5.31 mL/min/1.73 m^2^/year). Therefore, some patients with transplantation vintage of 10 years or longer benefitted from renal biopsy through treatment modifications, resulting in graft survival prolonged by 4 years.

Patients with IS enhancement showed significantly higher g, ptc, and cg scores than those without. The scores are reported to be prognostic factors [18–21] and can be used to diagnose antibody-related rejection (ABMR) [22]. The Banff classification defines g and cg scores as active and chronic scores, respectively. Furthermore, active glomerulitis is described to progress to transplant glomerulopathy (TG) [23, 24]. Among the scores, only the g score was significantly associated with improved eGFR slope after biopsy. Moreover, among those with positive g scores, the ones with mild chronicity (cg ≤ 1) experienced greater improvement in eGFR than those with severe chronicity (cg ≥ 2). The findings overall suggested that active glomerulitis without chronic TG can be treated with IS, even long after transplantation. This was in line with the fact that once TG is established, the lesion resists various treatments. In randomized placebo-controlled clinical trials, intravenous immunoglobulin plus rituximab [25] and bortezomib [26] failed to improve eGFR or ABMR features in patients with TG or late ABMR, respectively. In contrast, cg1a lesions are potentially treatable. This lesion is defined as an early glomerular membrane duplication, detected only by electron microscopy, in the Banff classification [12] and is known as the very early stage of TG. Indeed, patients with mvi and cg1a lesions developed TG within 18 months without treatment, whereas those treated with IVIg and rituximab, with or without plasmapheresis, did not develop overt TG for up to 4 years [27].

Stringer D et al. reported that interventions to improve adherence and optimize immunosuppression did not delay renal transplant failure after the development of DSA in a prospective, randomized, multicenter study [28]. In our study, interventions were based on kidney biopsy results, whereas their study relied on HLA antibody testing for interventions. Therefore, the findings of Stringer et al. do not negate our conclusion on the clinical significance of biopsies. Although our study was observational, it suggests that biopsies may provide better assessments of underlying pathology in transplant patients than clinical information alone.

The lack of DSA testing and C4d staining did not allow us to diagnose ABMR accurately in patients with microvascular inflammation. Among patients with intensified immunosuppression, 48.3% had mvi score of 2 or higher. Since not all of them had ABMR, borderline changes for TCMR or recurrent glomerulitis (immunoglobulin A nephropathy; IgAN) might explain the observed therapeutic effect of immunosuppressant enhancement.

It was reported that the recurrence of IgAN after renal transplantation is an important cause of graft failure [29]. No established therapy is currently available for recurrent IgAN. However, some Japanese researchers reported the efficacy of tonsillectomy with or without methylprednisolone pulse therapy for recurrent IgAN [30–33]. These studies show that tonsillectomy has improved hematuria, proteinuria, and pathological manifestations. In our study, seven patients underwent tonsillectomy after the indication biopsy. The improvement in eGFR slope in the entire group of seven individuals was not evident; however, there was a single notable case demonstrating significant improvement.

In this study, a higher percentage of patients received CsA than tacrolimus (TAC), since many patients underwent transplantation before 2000. Previous randomized controlled trials had shown that TAC is associated with less rejection [34–36], better graft function, and better graft prognosis than CsA [34]. Since most of the patients in our study had received CsA with underuse of MMF (approximately 50%), the eGFR slope improvement with the addition of immunosuppressive agents may be attributed to inadequate IS before biopsy.

CNI toxicity is a common cause of allograft injuries. In the current study, more than 80% of patients had positive aah scores, a characteristic of CNI toxicity. Moreover, no improvement in the eGFR slope was observed in patients for whom the CNI doses were reduced. This may be due to the irreversible nature of CNI toxicity. In line with our study, the prevalence of arterial hyalinosis in a 10-year graft biopsy was as high as 80%–90% in patients receiving CsA [37, 38]. Arterial hyalinosis due to CNI toxicity has several effects on allografts. Blood flow in arteries with severe hyalinosis due to CNI toxicity has been reported to be approximately 20% of that in normal arteries [38], leading to interstitial fibrosis. Indeed, associations between the duration of CsA administration and graft loss/poor graft function had been reported previously [31]. The prevalent interstitial fibrosis observed in our study (∼70% with positive ci scores), probably due to CNI, can explain the steepness of eGFR slopes (∼−3 mL/min/1.73 m^2^/year) after biopsy even in patients with treatment modification.

The current study has several limitations. First, DSA measurement and C4d staining were not performed in most cases. This was because most biopsies are performed before insurance coverage of DSA measurements in Japan. Second, the histological improvement after treatment modification was not re-evaluated. Therefore, we could not discuss the relationship between various treatment modifications and histological alterations. Third, the usefulness of the Japanese standard eGFR formula used in this study has not been evaluated yet in relation to renal prognosis in Japanese kidney transplant recipients. Given that the estimation of glomerular filtration rate is affected by differences in creatinine generation among ethnicities, it would not be appropriate to apply the CKD-EPI equation to our study population, which consists predominantly of Japanese patients.

In conclusion, our study demonstrated the clinical significance of renal biopsy performed long after transplantation. Even in the chronic phase, biopsy results changed the treatment strategy in nearly half of the patients. IS enhancement led to an improved eGFR slope, indicating that a substantial proportion of patients experience latent rejection. This disagrees with the perspective of the Kidney Disease: Improving Global Outcomes (KDIGO) guidelines, which suggest a gradual reduction of IS owing to the adaptive responses of the immune system in KTRs towards foreign antigens within the graft [39].

## Data Availability

The raw data supporting the conclusions of this article will be made available by the authors, without undue reservation.

## Abbreviations

ABMR, antibody-related rejection; ANOVA, analysis of variance; CI, confidence interval; CNI, calcineurin inhibitor; CsA, cyclosporine A; eGFR, estimated glomerular filtration rate; HLA, human leukocyte antigen; IgAN, immunoglobulin A nephropathy; IQR, interquartile range; IS, immunosuppression; IFTA, interstitial fibrosis and tubular atrophy; KDIGO, Kidney Disease: Improving Global Outcomes; KTR, kidney transplant recipient; MMF, mycophenolate mofetil; PSL, prednisolone; TAC, tacrolimus; TG, transplant glomerulopathy; UACR, urine albumin-to-creatinine ratio.
