# Supplementary material for: Clinical Impacts of Allograft Biopsy in Renal Transplant Recipients 10 Years or Longer After Transplantation
Source: Transpl Int. 2024 Jul 18;37:13022. doi: 10.3389/ti.2024.13022 (PMC11292417; doi:10.3389/ti.2024.13022)
Supplement: Supplementary file 1 [file Table1.docx]

**Supplemental Table 1. Treatment modifications after graft biopsy in 51 patients**

| Intervention after graft biopsy | n (%) |
| --- | --- |
| Enhancing immunosuppression | 30 (30) |
| Methylprednisolone pulse therapy | 12 (12) |
| 15-deoxyspergualin | 9 (9) |
| Increase in dose of any immunosuppressants | 9 (9) |
| Dose reduction of CNI | 10 (10) |
| Change of immunosuppressant agents * | 4 (4) |
| Tonsillectomy | 7 (7) |
| No change of treatment | 48 (49) |
