# Supplementary figures and images for: Clinical Impacts of Allograft Biopsy in Renal Transplant Recipients 10 Years or Longer After Transplantation
Source: Transpl Int. 2024 Jul 18;37:13022. doi: 10.3389/ti.2024.13022 (PMC11292417; doi:10.3389/ti.2024.13022)

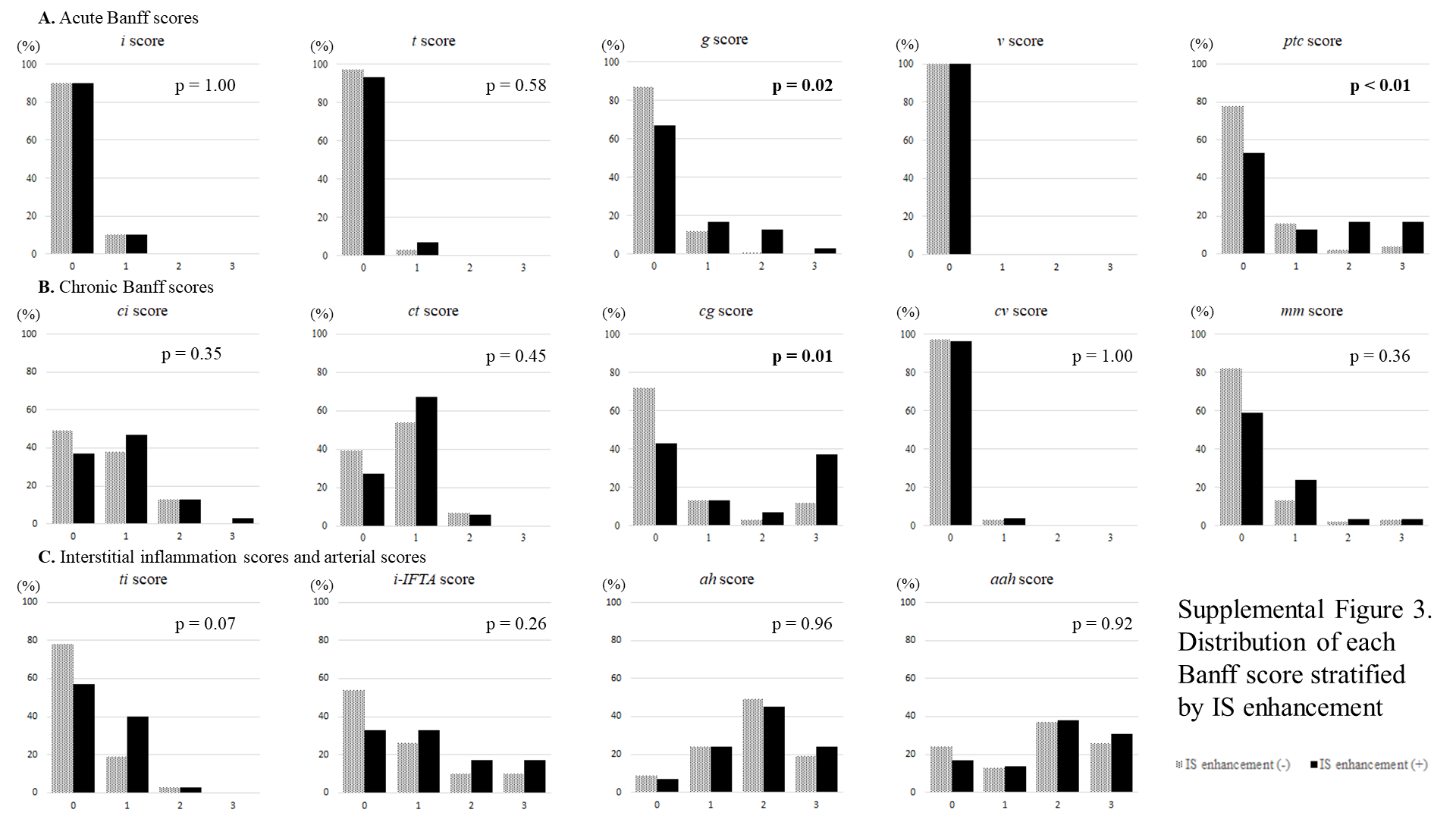

Supplement: Supplementary file 2 [file Image3.tif]

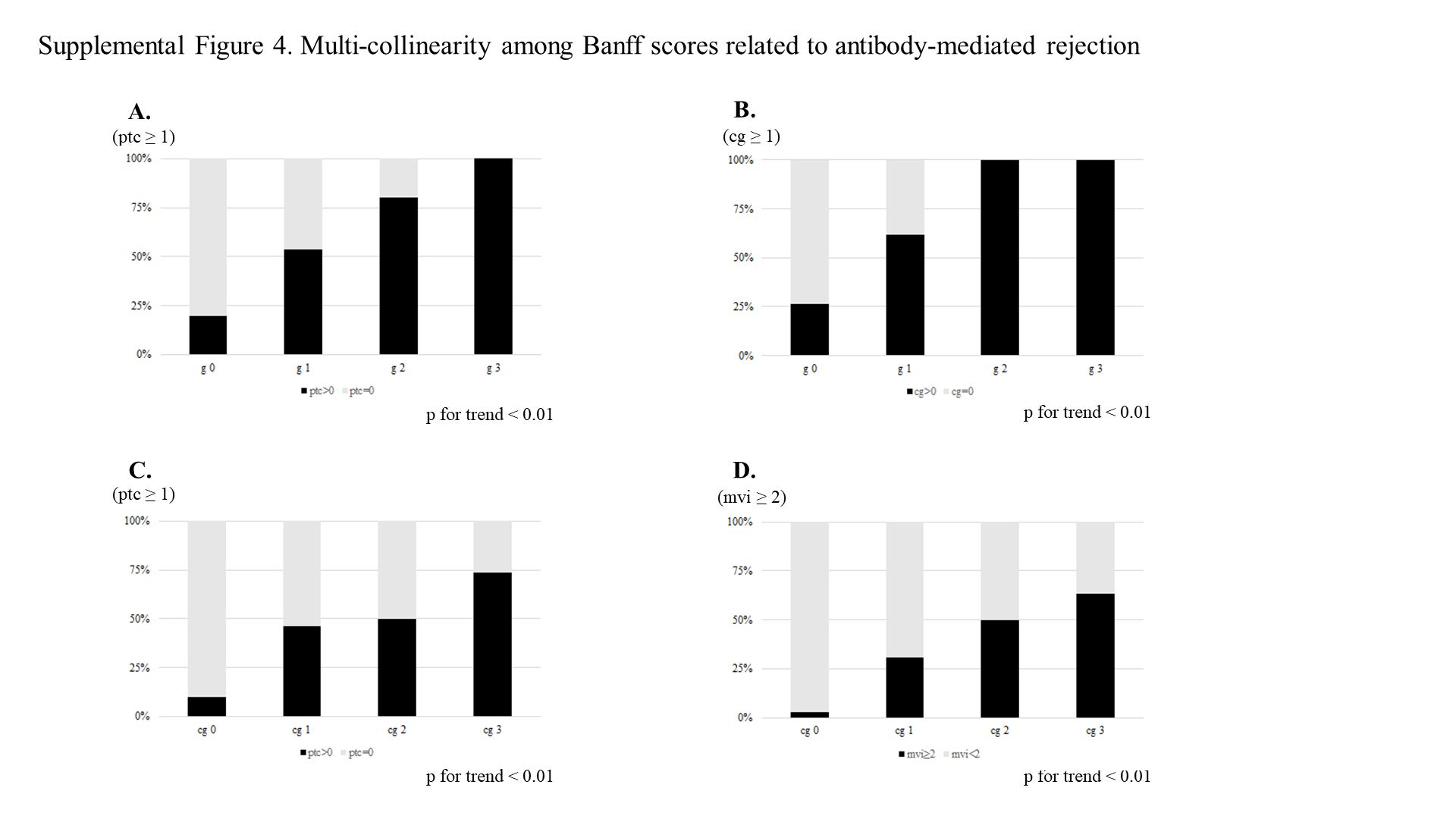

Supplement: Supplementary file 3 [file Image4.tif]

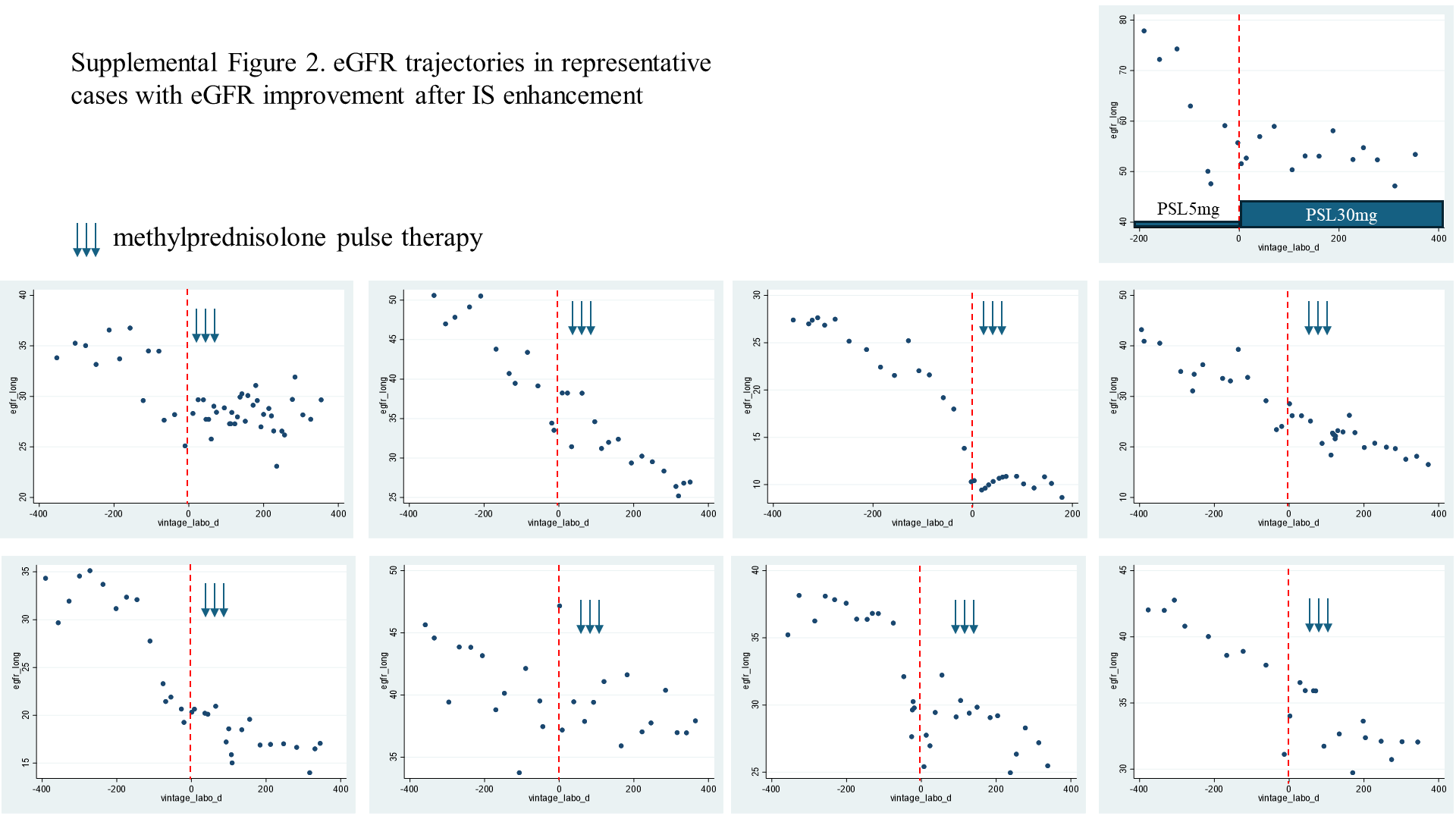

Supplement: Supplementary file 4 [file Image2.tif]

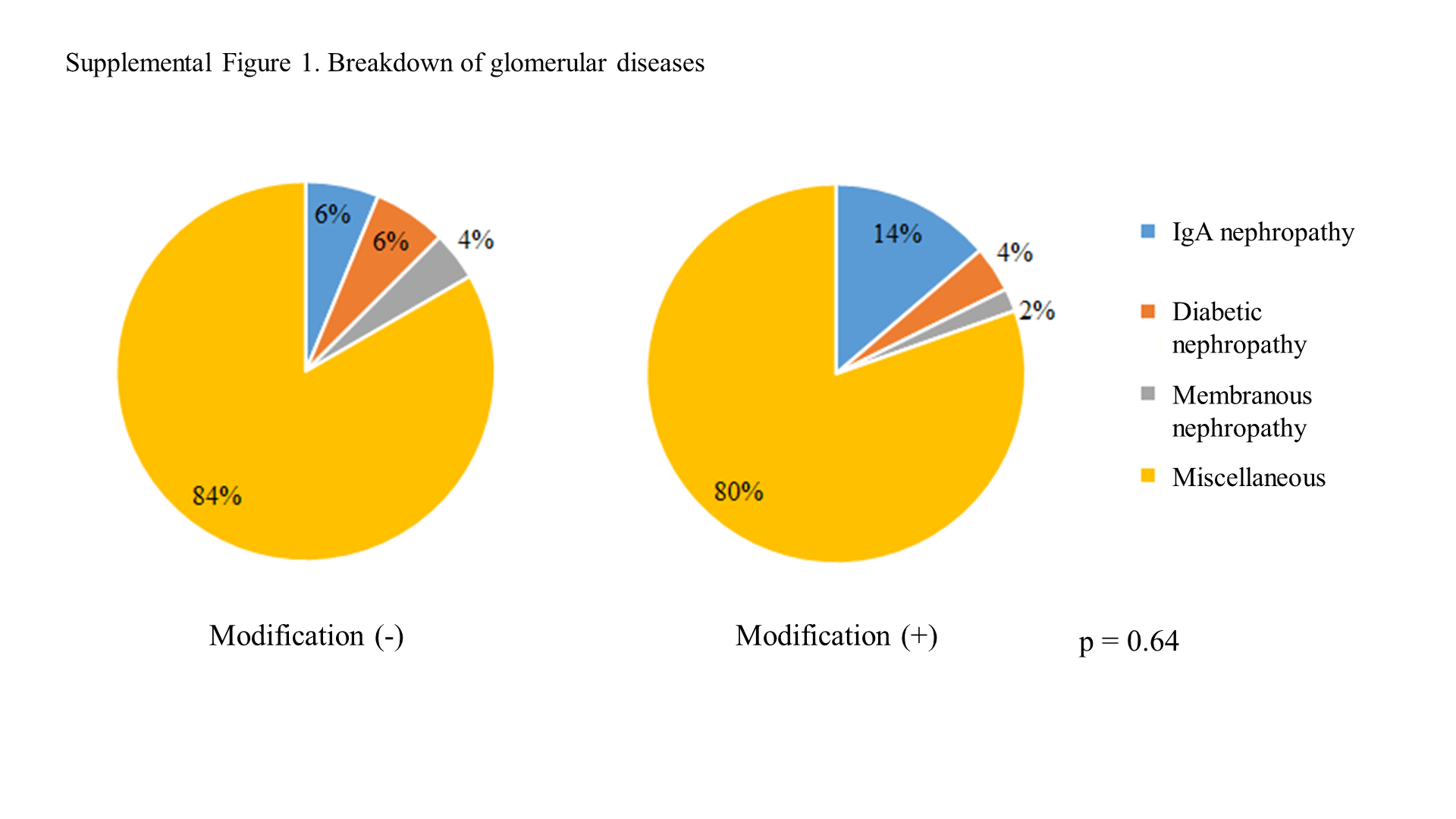

Supplement: Supplementary file 5 [file Image1.tif]
