# Supplementary material for: Clinical Impacts of Allograft Biopsy in Renal Transplant Recipients 10 Years or Longer After Transplantation
Source: Transpl Int. 2024 Jul 18;37:13022. doi: 10.3389/ti.2024.13022 (PMC11292417; doi:10.3389/ti.2024.13022)
Supplement: Supplementary file 6 [file DataSheet1.docx]

**Supplemental Figure 1. Breakdown of glomerular diseases**

Breakdown of glomerular diseases in patients with no treatment modification (A) and in those with treatment modification (B).

**Supplemental Figure 2. eGFR trajectories in representative cases with eGFR slope improvement after IS enhancement**

The panels show attenuation of the eGFR slope after index biopsy in patients receiving increased immunosuppressants based on the biopsy results. Time 0 denotes the time of index biopsy.

Abbreviations: eGFR, estimated glomerular filtration rate; IS, immunosuppression, PSL, prednisolone

**Supplemental Figure 3. Distribution of each Banff score stratified by IS enhancement**

Patients with IS enhancement had significantly higher g, ptc, and cg scores than those without enhancement.

Abbreviation: IS, immunosuppression

**Supplemental Figure 4. Multi-collinearity among the Banff scores related to antibody-mediated rejection**

Significant associations were observed between the g and ptc scores (A), g and cg scores (B), cg and ptc scores (C), and cg and mvi scores (D).

**Supplemental Table 1. Treatment modifications after graft biopsies in 51 patients**

For mutual exclusivity, major treatment modifications were adopted for patients with multiple modifications. For example, patients receiving methylprednisolone pulse therapy with changes in immunosuppressant agents were categorized as having IS enhancement.

Abbreviations: CNI, calcineurin inhibitor; IS, immunosuppression

**Capsule Sentence Summary**

The allograft indication biopsy results of 99 long-term kidney transplants (>10 years) altered treatment in nearly half of the patients. Intensified immunosuppression based on biopsy results improved eGFR slope, especially in patients with active glomerulitis without chronicity (transplant glomerulopathy).

(39 words)
